# Simultaneous Bilateral Avascular Necrosis of the Femoral Heads Associated With Cocaine Use

**DOI:** 10.7759/cureus.9865

**Published:** 2020-08-19

**Authors:** Christine EL-Yahchouchi, Mohamad K Moussa, Zaynab Khalaf, Charbel D Moussallem

**Affiliations:** 1 Anesthesiology, American University of Beirut Medical Center, Beirut, LBN; 2 Orthopedic Surgery, Lebanese Hospital Geitaoui, University Medical Center, Beirut, LBN; 3 Endocrinology and Diabetes, Lebanese University Faculty of Medicine, Beirut, LBN

**Keywords:** avascular necrosis, hip, primary hip replacement, cocaine

## Abstract

We present a case of a 38-year-old female patient, presenting with debilitating simultaneous bilateral avascular necrosis of the femoral head (AVNFH) 10 years after cocaine detoxification, making her wheelchair-bound for six months. This case is reported for the rarity of association of cocaine with AVNFH, and for the unique fact of the simultaneous bilateral condition occurring a long time after cocaine ingestion in the absence of other important risk factors.

This report postulates cocaine as a possible cause of bilateral AVNFH, which can increase the index of suspicion of this pathology, allowing early diagnosis and better outcomes.

## Introduction

Osteonecrosis, aseptic necrosis, and ischemic necrosis are all known nomenclature of the same multifactorial pathologic process of the avascular necrosis of the femoral head (AVNFH). The most common causes mentioned in literature for this condition are two groups: atraumatic, mostly related to steroids and alcohol, and traumatic disruption of the vascular supply to the femoral head [1].

The relation between cocaine and AVNFH is not well-established, with rare cases describing it. To the best of our knowledge, there is only one case report about cocaine-induced unilateral AVNFH published in 2017 by Li et al., where the patient also had other associated risk factors [2].

We present herein a rare case of simultaneous bilateral AVNFH in a young female patient, 10 years after cocaine detoxification. This report adds more data to the literature about cocaine-induced AVNFH, which would facilitate the understanding of this condition, allowing early diagnosis and better outcomes.

## Case presentation

This was a 35-year-old female patient, mother of three girls, known to have pseudo-seizures on anxiolytic therapy, presenting to the clinic, bound to a wheelchair for six months prior to presentation, complaining of severe bilateral hip pain preventing her from ambulation.

She was self-treated by over-the-counter medication with pain killers and non-steroidal anti-inflammatory drugs without improvement.

Her past medical history was significant for cocaine ingestion 10 years ago, after which she underwent a successful detoxification program. She denied having any chronic illnesses and did not receive steroid treatment throughout her life.

**Figure 1:**
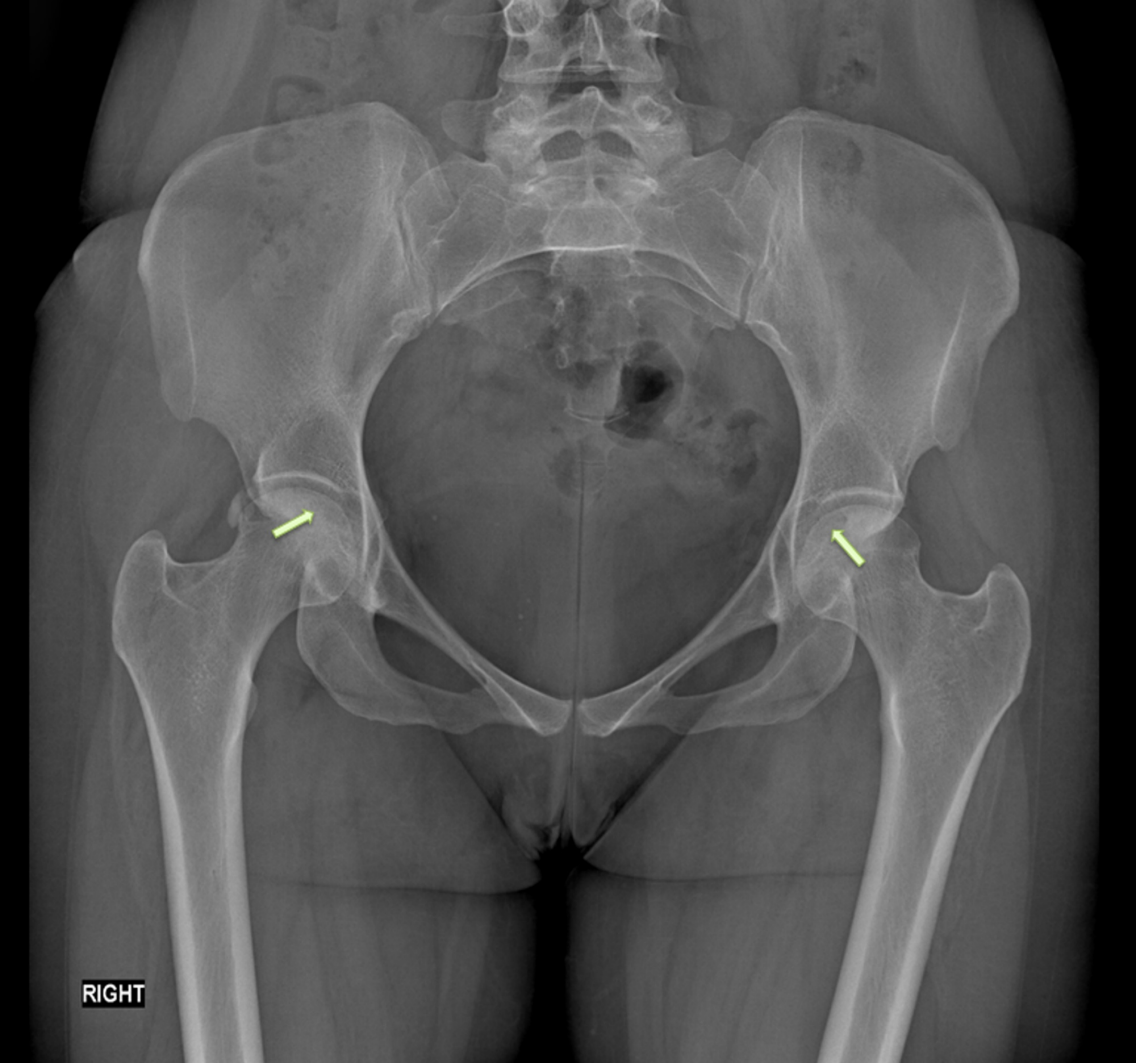
AP radiograph of the pelvis showing crescent signs AP: anteroposterior

**Figure 2:**
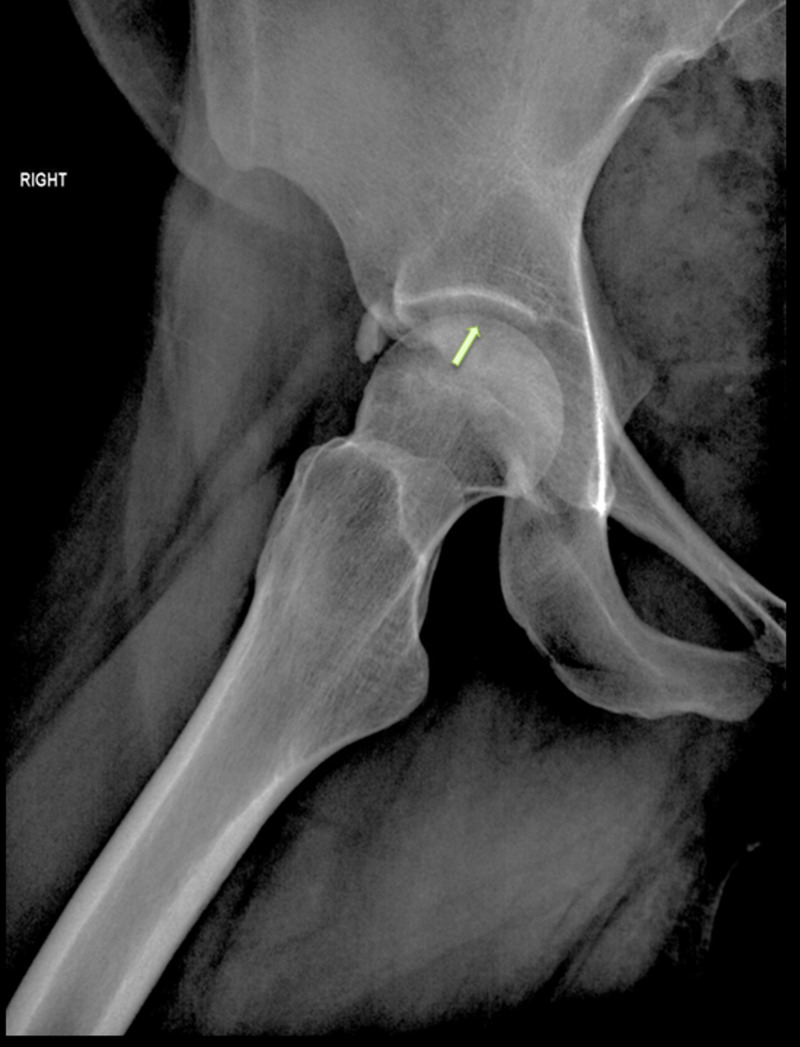
Lateral radiograph of the right hip showing a crescent sign

**Figure 3:**
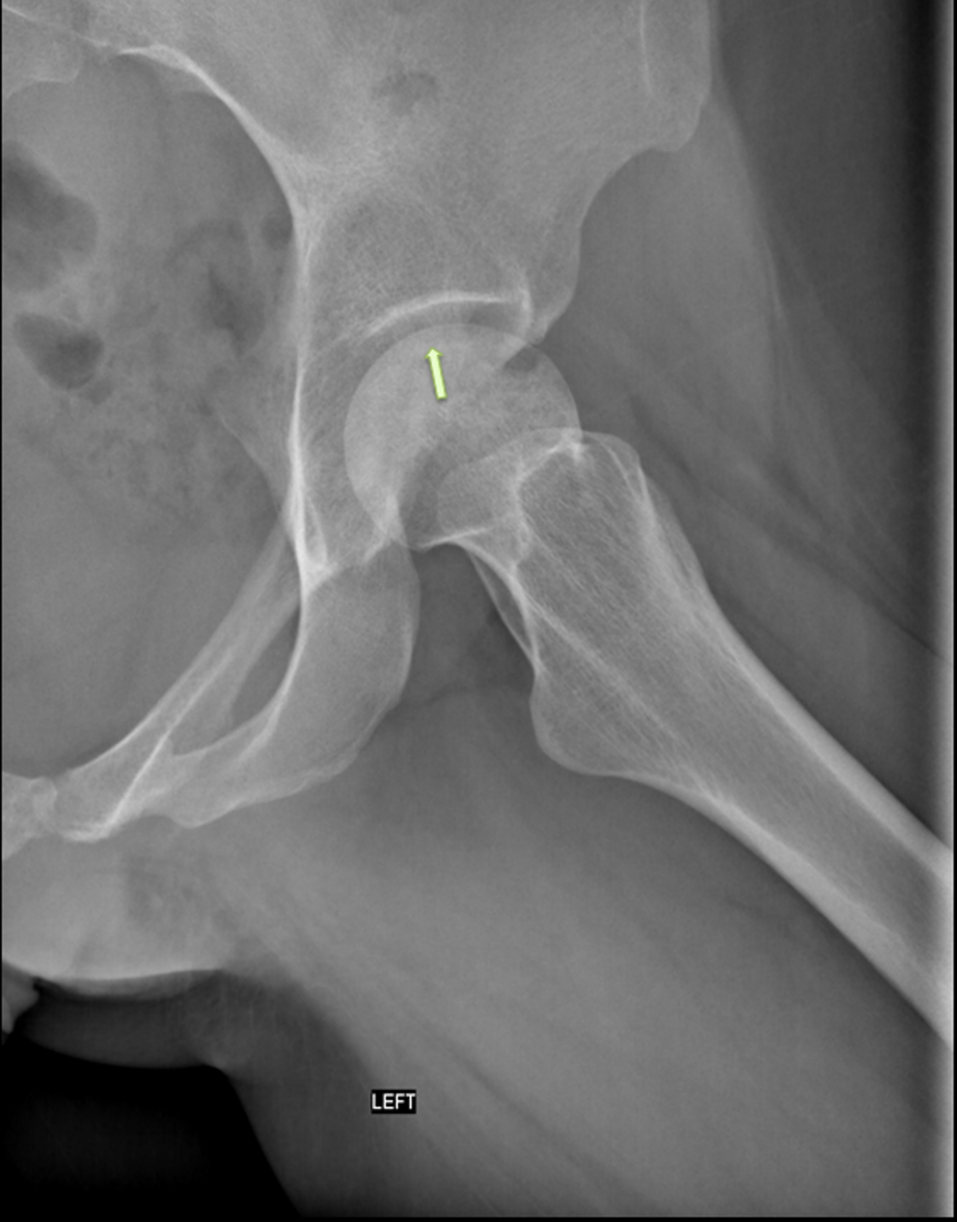
Lateral radiograph of the left hip showing a crescent sign

**Figure 4:**
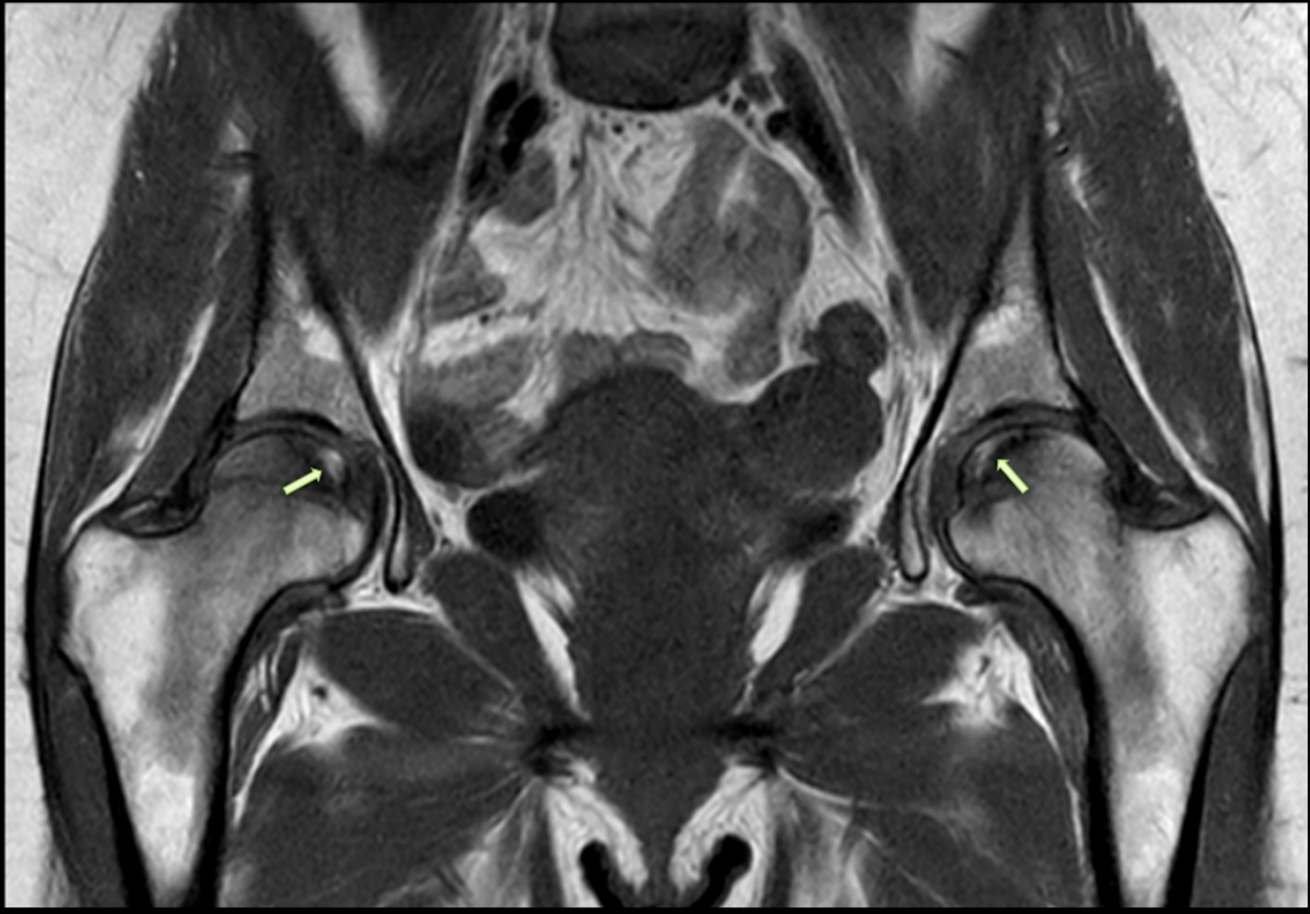
Coronal T2-weighted MRI of the pelvis showing bilateral AVNFH AVNFH: avascular necrosis of the femoral head; MRI: magnetic resonance imaging

**Figure 5:**
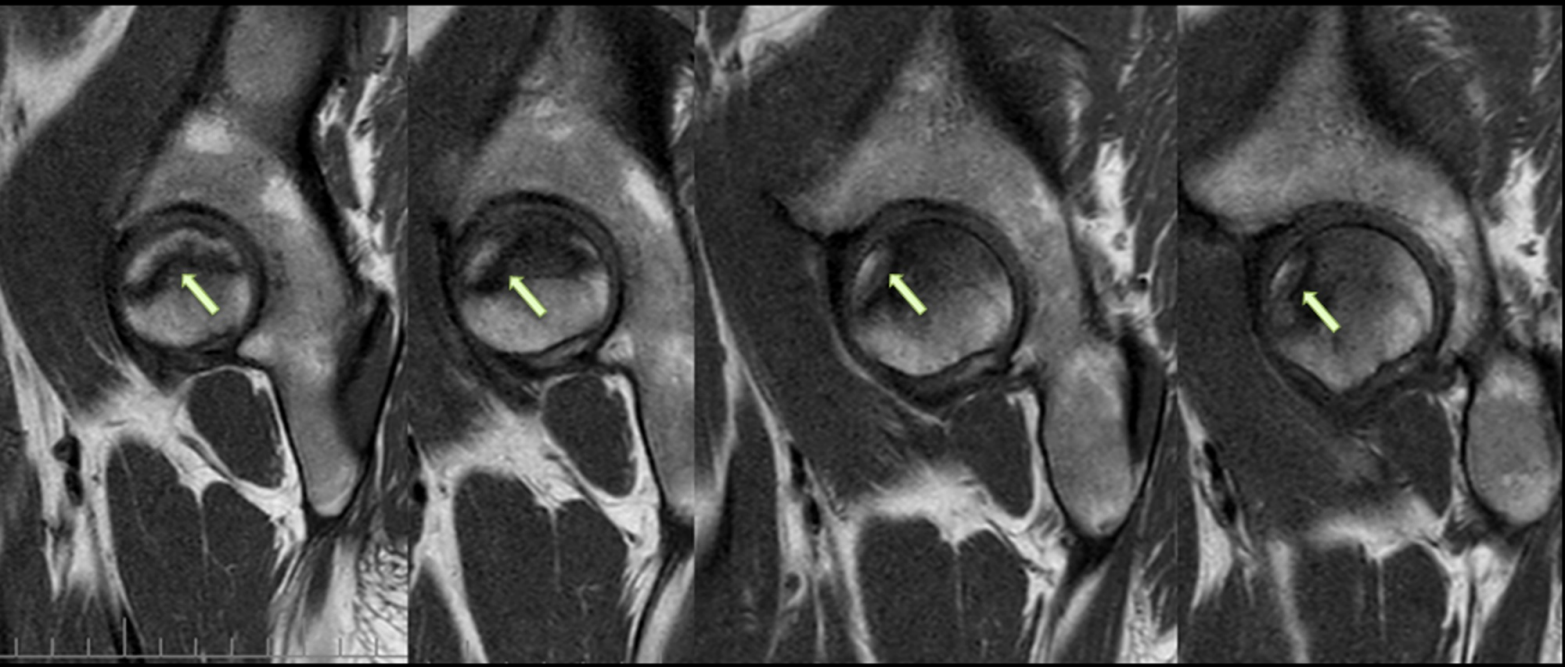
Sagittal T2-weighted MRI of the pelvis showing AVNFH AVNFH: avascular necrosis of the femoral head; MRI: magnetic resonance imaging

On physical examination, the patient was barely able to stand; her pain was more significant on the right side. Flexion of both hips was painful but without limitation in range of motion except for a slight limitation of internal rotation that is best seen on hip flexion. She was also having severe weakness in the abductors. Her initial radiograph and MRI are shown in Figures 1-5.

She was diagnosed with bilateral grade II-a avascular necrosis according to the Ficat and Arlet classification [3].

Full laboratory workup was done, including complete blood count with differential, erythrocytes sedimentation rate, c-reactive protein, creatinine, liver panel, lipid panel, and serology for sexually transmitted diseases. They all turned to be within normal limits.

Facing the patient’s severe debilitating state and due to her functional incapacity and social withdrawal, she was planned for bilateral total hip replacement in a two-step procedure separated by a six-week period.

**Figure 6:**
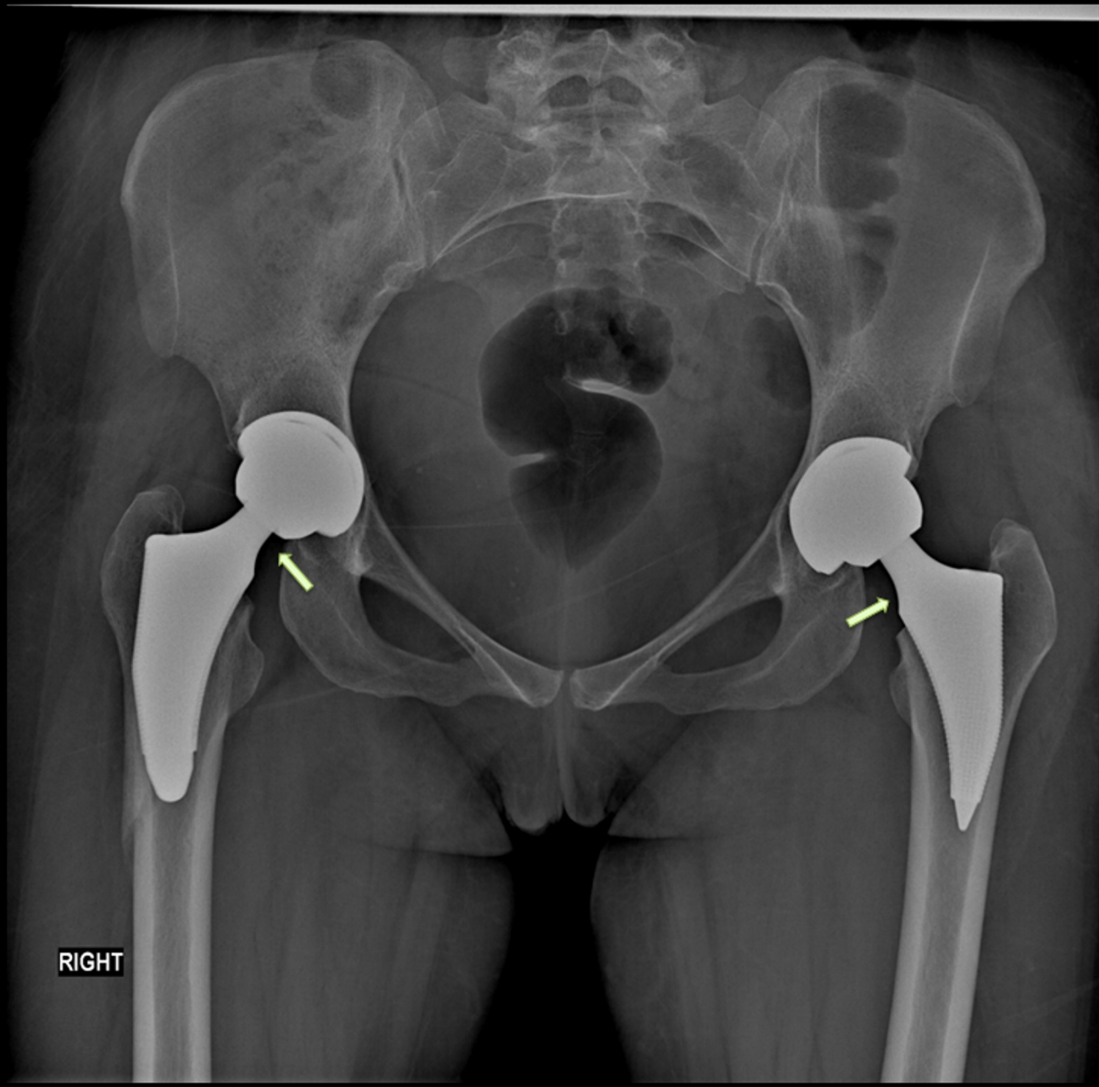
Postoperative radiograph of the pelvis showing bilateral total hip ceramic on ceramic replacement

Short-stem ceramic on a ceramic liner was chosen and total hip arthroplasty was performed bilaterally in two different times. Figure 6 shows the final postoperative radiographs after the second operation. The pathology results of both femoral heads confirmed the diagnosis of AVNFH with aseptic necrosis and dystrophic bone.

The patient was first satisfied by the result and she returned to her daily activity but, unfortunately, she had two episodes of prosthetic dislocation on the left side that was attributed to the weakness of the abductor muscles due to preoperative bedridden status. She was successfully treated with closed reduction. Eighteen months after surgery, the patient is now satisfied with the result and has a pain-free normal gait.

## Discussion

AVNFH is a joint-threatening condition that may lead to joint destruction, where 80% of untreated patients will develop head collapse and 40% to 70% will develop AVNFH on the other side [4]. It accounts for 10% of total hip arthroplasties in the United State [5] and is the most common cause of total hip arthroplasty in young patients with an average age between 33 and 38 [4].

Its exact prevalence is unknown, but approximately 20,000 to 30,000 new patients are diagnosed each year in the United State [6]. No typical presentation is present for AVNFH; non-specific hip pain, with sometimes irradiation above the knee, is the most common presentation. Physical examination may be relevant for the decreased internal rotation that is more prominent on flexion (sectoral sign or axis deviation test). [7] Therefore, a high index of suspicion and careful history should be present in order to make an early diagnosis.

Several mechanisms have been proposed to elicit the pathophysiology of AVNFH.

The most adopted theory is the alteration of blood to the femoral head [6]. It is thought that compromised subchondral microcirculation ultimately leads to necrosis of the head with subsequent accumulation of microfractures that will end up with head collapse [8].

This vascular compromise can be due to intravascular coagulation or extravascular compression. Intravascular coagulation results from the formation of thrombus in hypercoagulable states in sickle cell anemia (SCA), thrombophilias, antiphospholipid syndrome (APLS), malignancy, and inflammatory bowel disease (IBD) or from microemboli such as in fat embolism (FE) and nitrogen embolism (Caisson disease (CD) [9].

However, extravascular compression results from nutrient vessel injury leading to the accumulation of blood and fat in the extravascular space, which ends up causing extravascular compression [4]. This is seen in hip fracture and dislocation (mostly femoral neck fracture) [10].

Another theory to be added is the altered bone physiology, where impaired mesenchymal cell differentiation leads to increased adipogenic volume, decreased osteogenesis, and altered bone structure. This can be caused by endogenous factors, such as genetic mutation of collagen type II [11], or by exogenous factors such as alcohol [12].

Steroids administration is proven to be one of the most common causes of AVNFH, increasing the risk by 20 times [13], multiple mechanisms (vasoconstriction) [14], intraosseous venous stasis, and secondary extravascular compression [15], fat microemboli [16], and alteration of bone physiology [17].

Furthermore, hyperlipidemia (HL) has been shown to act on multiple pathways, where patients with HL have increased risk of thrombus formation and high FE risk (intravascular obstruction), endothelial dysfunction (extravascular compression), and increased adipogenesis and bone marrow pressure (alteration of bone physiology) [18].

To add, Gaucher disease (GD) is a rare cause that has been associated with AVNFH by acting on all the pathophysiologic mechanisms mentioned above.

**Figure 7:**
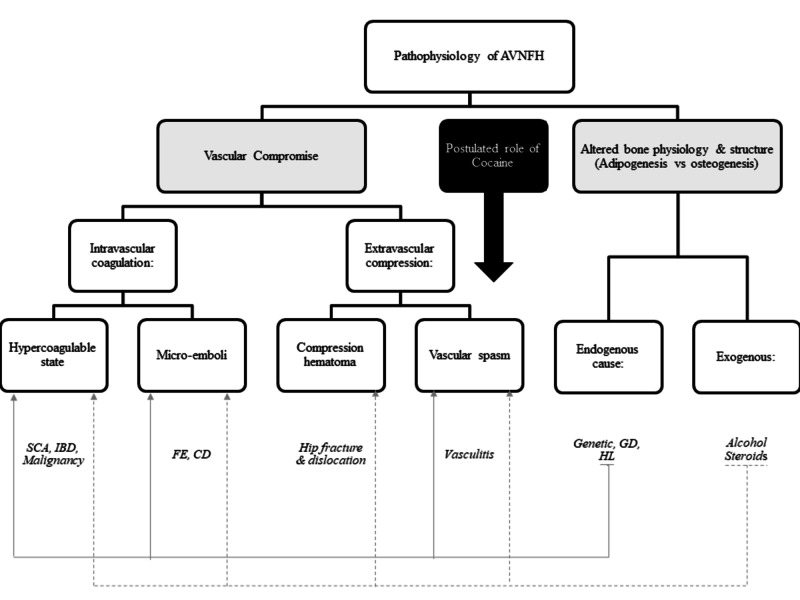
Summary of the pathophysiological pathways and etiological factors listed in our review with the postulated role of cocaine in the pathogenesis of AVNFH AVNFH: avascular necrosis of the femoral head

Figure 7 represents a summary of the pathophysiological pathways and etiological factors listed above with the postulated role of cocaine in the pathogenesis of AVNFH.

## Conclusions

AVNFH, a condition that we like to call bony compartment syndrome, is still ambiguous in terms of etiological factors. Its most common cause is still labeled as idiopathic. This case report suggests cocaine as a new risk factor or probably a new causative agent, of osteonecrosis in the lower limb, specifically the femoral head. With the increasing level of cocaine use, we suggest that physicians are extra-vigilant in any patient complaining of hip pain with a history of cocaine ingestion even after detoxification. This case report would increase the index of suspicion of AVNFH, allowing early diagnosis and better outcomes in patients.
